# Value-Added Dietary Fiber Concentrate Obtained as Waste after Protein Isolation from Ethanol-Treated Sunflower Meal

**DOI:** 10.1155/2022/4289059

**Published:** 2022-09-30

**Authors:** Petya Ivanova, Hristo Kalaydzhiev, Anton Slavov, Vesela I. Chalova

**Affiliations:** ^1^Department of Biochemistry and Molecular Biology, University of Food Technologies, 26 Maritsa Blvd, Plovdiv 4002, Bulgaria; ^2^Department of Analytical Chemistry and Physical Chemistry, University of Food Technologies, 26 Maritsa Blvd, Plovdiv 4002, Bulgaria; ^3^Department of Organic Chemistry and Inorganic Chemistry, University of Food Technologies, 26 Maritsa Blvd, Plovdiv 4002, Bulgaria

## Abstract

Deproteinized sunflower meal (DSM) was obtained as waste from ethanol-treated sunflower meal after alkaline extraction of proteins. The study aimed at biochemically and functionally characterizing the material concerning its potential practical application and valuability. The DSM consisted mainly of proteins (19.88%) and dietary fibers (61.06%) the majority of which were insoluble (53.09%). Cellulose (30.87%) and lignin (21.79%) were the most contributing compounds to the total amount of dietary fibers. The DSM contained Fe (133.29 mg/kg), Zn (201.56 mg/kg), and Cu (31.87 mg/kg). The analyses defined the DSM as a fiber concentrate with relatively high thermal stability. The distraction of the material began at 170°С with a maximum speed at 277°С. The highest water absorption capacity (WAC) of the DSM was observed at pH 6 and 7 (approximately 8 g H_2_O/g sample) under all studied conditions including pH from 3 to 10 and three levels of NaCl concentrations (0.00 M, 0.03 M, and 0.25 M). At pH 7, increasing temperature from 20°C to 60°C increased the WAC of the DSM from 8.13 g H_2_O/g sample to 9.80 g H_2_O/g sample, respectively. Further increase in the temperature diminished the WAC of the DSM. At pH 6, the increase in temperature did not influence positively the WAC of the DSM. The study demonstrated the potential of the DSM, a waste obtained from the protein isolation process, as a valuable ingredient/additive in the food industry.

## 1. Introduction

Sunflower meal is generated in substantial amounts as a by-product of the oil-producing industry [1]. According to the same author, the quantity of generated sunflower meal approximates 36% of the mass of the processed seed. Being rich in proteins, it is mainly used as a feed ingredient aiming to complementation of deficient amino acids. The application of this by-product in the feed industry, however, is reduced by the presence of some antinutrients such as phytic acid, tannins, saponins, chlorogenic acid, and high fiber content [2, 3]. Limited inclusion levels in livestock nutrition generate excessive amounts of sunflower meal which turns into waste when incompletely used. Dissipating industrial by-products or agricultural biological waste contradicts the concept of circular bioeconomy which was introduced by the EC [4] to face nowadays' societal challenges [5]. Steadily growing human population and natural resource overuse triggered concerns regarding long live sustainability of high living standards. It insistedn the movement of human society to a conceptually different way of thinking and organization of industrial technologies to respond to environmental awareness. As stated by Aschemann-Witzel and Stangherlin [5], a sustainable circular economy requires reinsertion of the biological waste and by-products into a value chain by their waste-free conversion into useful products. In addition to proteins, sunflower meal is rich in minerals, fibers, and phenolic compounds [6]. Thus, a potential upcycling of the sunflower meal could result in the preparation of multiple products with added value for the food and nutraceutical industries or agriculture. In general, the presence of phenols lowers the value of the sunflower meal as a feed ingredient or a raw material for protein isolation due to interference with protein palatability and digestibility [7]. Due to the high content of phenols in the sunflower meal [8]), their reduction can turn into a successful approach for the preparation of phenol-rich products with valuable functional properties while providing suitable material for protein isolation [9–11].

Numerous approaches exist for the preparation of protein isolates from meals [12]. They mainly differ by the type of the extracting agents and conditions including pH, temperature, solid to solvent ratio, and longevity of the process. Regardless of their diversity, the extinction of protein remains solid which is generally considered waste. However, hypothesizing potential usefulness, Georgiev et al. [13] established that the residual waste after protein isolation from ethanol-treated rapeseed meal was a fiber concentrate possessing physicochemical properties for functional food systems formulation. The current study aimed at the evaluation of the solid remaining as waste after protein isolation from ethanol-treated sunflower meal. The chemical composition, thermal stability, and water absorption capacity of the deproteinized sunflower meal residue (DSM) were investigated to evaluate the suitability of the material for a potential practical application.

## 2. Materials and Methods

### 2.1. Preparation of Deproteinized Ethanol-Treated Sunflower Meal Residue

**Figure 1:**
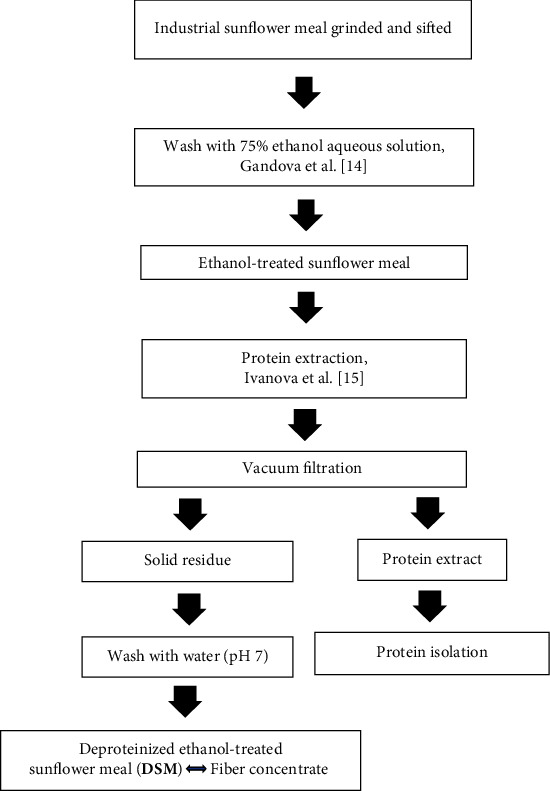
Preparation of deproteinized ethanol-treated sunflower meal.

The procedure consists of several steps outlined in Figure 1. Industrial sunflower meal, obtained from a local company, was ground and sifted to particles with a size ≤0.315 mm. To reduce phenol content, a sample was treated with 75% aqueous ethanol solution 4 times [14]. Proteins were extracted from a 5% (w/v) ethanol-treated sunflower meal suspension (pH 12) at 40°C for 60 min under continuous agitation [15]. The solid residue was separated by vacuum filtration and washed with distilled water (pH 7) several times to reach neutral pH. The deproteinized ethanol-treated sunflower meal, namely DSM, was air-dried at room temperature and stored in closed containers for further analyses. The protein extract was used for protein isolation and characterization in a separate study.

### 2.2. Biochemical Analyses

Protein was quantified by the Bradford method [16] after solubilization of a DRM sample in 0.5 N NaOH for 15 min under boiling. Bovine serum albumin was used for the preparation of a standard curve. The amount of total lipids was evaluated as described by Bligh and Dyer [17], while ash content was determined by ICC Standard №104/1 [18]. Phenols were extracted with 70% aqueous ethanol solution as described by Petkova et al. [19] and quantified by using Folin–Ciocalteu reagent [20]. Selenium (Se) was determined by using inductively coupled plasma optical emission spectrometry (ICP-OES) [21]. A Bulgarian national standard procedure (BDS) [22] was used for the evaluation of the other microelements and heavy metals studied.

Total dietary fibers (soluble and insoluble) were determined according to AOAC method [23] using a Total Dietary Fiber Assay kit (K-TDFR-100А, Megazyme, Ireland). Total (noncellulosic) carbohydrates, uronic acids, cellulose, and lignin were evaluated from an alcohol-insoluble fraction of the DSM sample. The latter was obtained as described by Georgiev et al. [13]. The quantification of total (noncellulosic) carbohydrates, uronic acids, cellulose, and lignin was conducted as outlined by Escalada Pla et al. [24, 25]. Monosaccharide composition was evaluated by high-performance liquid chromatography (ELITE La Chrome, Hitachi High Technologies America, Inc., San Jose, CA, USA) after hydrolysis of DSM with 2 M CF_3_COOH as previously reported [26]. Contents were calculated on a dry matter basis unless otherwise specified.

### 2.3. Thermal Stability

The thermal stability of DSM was evaluated by thermal gravimetric analysis (TGA) and differential scanning calorimetry (DSC) tests as previously described [13]. Briefly, the TGA was conducted by heating the sample in the air from 25°С to 550°С with an increment of 5°С/min followed by cooling with 20°С/min till 25°С. The DSC test was implemented by heating a sample in nitrogen from 25°С to 180°С with an increment of 5°С/min and cooling the system at the same speed till 25°С. The TGA and DSC were performed with Q50 and Q20 TA instruments (New Castle, DE, USA), respectively.

### 2.4. Determination of Water Absorption Capacity (WAC)

WAC was determined as described by Rodriguez-Ambriz et al. [27]. A 100 mg sample with adjusted pH (3-10) was mixed with 1 mL distilled water with the same pH. Either HCl or NaOH was used to reach the needed pH of the sample and water. NaCl was added at two levels, 0.03 M and 0.25 M, whenever needed. The resulted suspension was vortexed (Advanced vortex mixer–ZX3, VELP Scientifica, Usmate (MB), Italy) for 30 s and incubated at room temperature for 30 min. It was further centrifuged for 20 min at 1800 × *g* (23°C) (MPW-251, Med. Instruments, Poland), and the supernatant was decanted for 10 min at an angle of 45°. The influence of temperature was evaluated at pH 6 and 7 in a wide range from 20°C to 90°C with an increment of 10. WAC was calculated by dividing the weight of the absorbed water (*g*) by the weight of the DSM sample (*g*).

### 2.5. Statistical Analyses

Analyses were performed in triplicates. Results are presented as means ± standard deviation (SD). Data were analyzed by one-way analysis of variance (ANOVA) using Statgraphics Centurion statistical program (version XVI, 2009) (Stat Point Technologies, Ins., Warrenton, VA, USA). Mean differences were established by Fisher's least significant difference test for paired comparison with a significance level *α* = 0.05.

## 3. Results and Discussion

### 3.1. Biochemical Characterization

Sunflower meal has been extensively studied as a source for the preparation of protein isolates [6, 28, 29], but little attention is paid to its fiber fraction and potential application. Although neglected as a valuable compound, the DSM appeared a rich source of proteins and fibers (Table 1). It contained 19.88% proteins and 61.06% dietary fibers, the majority of which were insoluble (53.09%, Table 1). The relatively high amount of proteins embedded in the residue may be due to the limited alkaline extractability of the proteins from industrially produced sunflower meal. After optimizing extraction conditions, Ivanova et al. [15] achieved a protein yield lower than 60% (pH 10). Combining pH 7.3 and the addition of NaCl (0.3 M), Slabi et al. [30] yielded 46.83% total protein with good solubility and functional properties. During seed processing and, further, protein extraction, proteins are subjected to numerous physicochemical modifications. Along with denaturation and conformational changes, the proteins are involved in multiple reactions leading to composite molecules with higher weight and low solubility. Some examples of these are Mallard products, resulting from protein-carbohydrate interactions [31], and protein-phenol complexes formed after the reaction between the two groups of compounds [32]. According to Sari et al. [33], the chemical composition of the biomass, used for alkaline extraction of the proteins, is crucial as the high levels of cellulose and oil decrease extraction efficiency. The fibrous concentrates prepared from defatted sunflower flour by extraction with bisulfite contained 60.84 g/100 g fiber and 35.67 g/100 g protein [34].

Sunflower seeds are rich in fibers [6] which amount increases after oil removal [35]. The ethanol treatment of the sunflower meal [14], followed by the alkaline extraction of the proteins, resulted in a product, namely DSM, containing 61.06% dietary fibers (Table 1). Dietary fibers are defined by the American Association of Cereal Chemists [36] as “the edible parts of plants or analogous carbohydrates that are resistant to digestion and absorption that include polysaccharides, oligosaccharides, lignin, and associated plant substances”. Having more than 50% dietary fiber along with proteins, lipids, minerals, and water, the DSM could be considered a dietary fiber concentrate [37]. In the text from now on, DSM and fiber concentrate will be interchangeably used to indicate the product. Many studies, clearly reviewed by He et al. [38], evidenced the positive effect of dietary fibers on human physiology. Consumption of food, rich in dietary fibers, decreases the incidence of chronic diseases such as diabetes, obesity, cancer, and intestinal disorders. Szajnar et al. [39] established that the addition of chokeberry fiber to sheep milk stimulated the growth of two different probiotic monocultures *L. acidophilus* and *L. rhamnosus* and affected the organoleptic properties of the fermented milk. Safdari et al. [40] reported a combined beneficial influence of banana fiber and banana peel fiber added to camel milk due to enhanced survival of probiotic bacteria, *Lactobacillus casei* and *Lactobacillus gasseri*, and reduced syneresis of the fermented product, i.e., yogurt. Modulation of physicochemical, textural, and sensory properties of yogurt and fruit jams by the addition of guar and bamboo fibers was reported by Barak and Mudgil [41] and Dordevic et al. [42], respectively. Containing valuable macroelements (Table 1), the DSM could be potentially used as a food additive with techno-functional properties and human health benefits which, however, should be further explored.

Besides, the presence of both proteins and fibers implies a potential application of the DSM as an ingredient for bioplastic fabrication. Bioplastics are renewable and biodegradable alternatives to petroleum-based products [43]. Since the mechanical properties of the material mixture depend on protein: fiber ratio [44], the DSM supplementation could be used to reach a blend with desired techno-functional characteristics. Water sorption capacity is also influenced by the quantities of these two compounds with fiber concentration being more contributing to this feature [44]. Water sorption, however, can be either desired or undesired process, and a simple modification of a food matrix by varying an ingredient concentration would be of practical interest.

Plant-derived dietary fibers are still known as cell wall carbohydrates [45]. Most of the fibers contained in the sunflower meal originated from seed hulls which remained as a part of the meal after oil extraction [46]. Dehulling sunflower seeds is not a standard stage in oil-producing technologies but was suggested by the same authors for achieving a high-protein sunflower meal. The DSM contained a high ratio (approximately 10) of insoluble (53.09%) to soluble fibers (4.59%) (Table 1). Cellulose (30.87%) and lignin (21.79%) were the most contributing compounds (Table 2) to the total amount of dietary fibers (Table 1). The cell wall polymers cellulose and lignin were reported as the main constituents of a dietary fiber concentrate prepared from papaya by-products [47]. Although less valuable for food-processing technologies, the insoluble fibers, because of their swelling properties, facilitate the transit of the ingested food to the small bowel and are thus considered beneficial for human health [38].

The HPLC analysis of monosaccharide content of the DSM demonstrated glucose as the compound in the highest amount (180.49 *μ*g/mg), followed by xylose (98.14 *μ*g/mg), and galactose (35.82 *μ*g/mg) (Table 3). Although with different values, the trend follows the one obtained by Georgiev et al. [13] for deproteinized rapeseed meal. Glucose and xylose were the monosaccharides in the highest amounts in the sunflower hydrolysate prepared by Bautista et al. [48] as well. The same authors demonstrated its suitability as a carbon source for solid-state fermentation with different fungi which implies a potential applicability of the DSM in the biofood industry.

Microelemental analysis revealed relatively high amounts of Fe (133.29 mg/kg), Zn (201.56 mg/kg), and Cu (31.87 mg/kg) in the DSM (Table 4) as the latter two were 3 and 4 folds higher than the corresponding microelements in deproteinized rapeseed meal [13], respectively. The DSM contained Se (0.03 mg/kg) but was still 5-fold lower than Se content in the deproteinized rapeseed meal (0.16 mg/kg). Regardless of the substantial amounts of physiologically important microelements in the DSM, the presence of other compounds in the product (Table 1) imposes their bioavailability evaluation before use for deficiency compensation. Substances such as proteins, fibers, and phenols may decrease the absorption of bivalent ions (Zn^2+^, Cu^2+^, Fe^2+^, and Mn^2+^) by their binding into indigestible complexes [49]. No heavy metals, Pb and Cd, have been established in the DSM.

### 3.2. Thermal Stability

**Figure 2:**
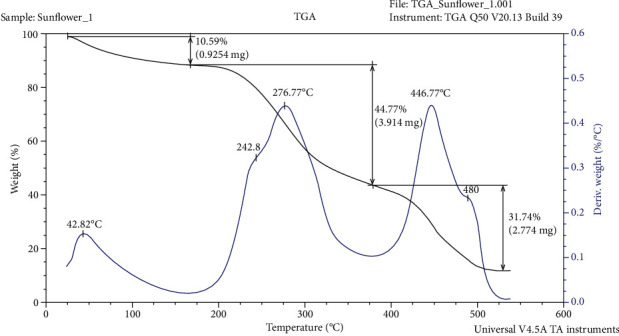
Thermal gravimetric analysis of deproteinized ethanol-treated sunflower meal.

Thermogravimetric analysis (TGA) and differential scanning calorimetry (DSC) were used to evaluate the thermal stability of DSM. The TGA demonstrated that the change in the weight of the sample in the temperature range 25-550°C occurred in three stages (Figure 2). Heating the sample from 25°C to 168°C resulted in a weight decrease of 10.6%, which was probably due to the release of water. The degradation of the material started after 170°С and happened in two stages: 170-379°С and 380-550°С. The increase of the temperature to 379° C decreased the sample wait by another 44.8%. The temperature range from 170 to 379°С was associated with thermal destruction of the material, as the maximum speed of the process was at 277°С. At this temperature interval, a second and smaller peak was observed with a maximum at 243°C. Most probably it was a consequence of disruption of chemical bonds with close energy which resulted in the release of products with different molecular weights. As reported by Hancock and Zografi [50], cellulose has a glass transition temperature of 226.85°C. Products with a composite nature as DSM though would have specific thermal characteristics defined by its qualitative and quantitative profile. By raising the temperature to 550°C, the sample weight was reduced by 31.7%. This stage was associated with oxidative destruction of a charcoal residue. A similar smaller peak was also observed here, with a maximum at 480°C. The peak oxidation rate was at 447°C. The solid residue remaining at 550°C was 12.9% (by weight) of the initial weight of the sample.

**Figure 3:**
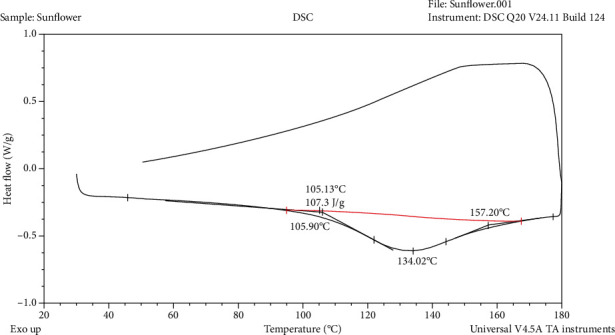
Differential scanning calorimetry of deproteinized ethanol-treated sunflower meal.

The DSC curve of the sunflower fiber concentrate, i.e., the DSM was characterized by a wide endothermic peak starting at 106°C and ending at 157°C with a maximum at134 °C (Figure 3). It is most probably due to the release of water. The heating from 30 до 180°С was characterized by enthalpy Δ*H* = 107 *J*/*g*, while the subsequent cooling of the sample to 50° C was not associated with thermal effects. The DSC measurements showed that a large part of the absorbed water (~10.6%) was probably strongly bound to cellulose molecules, so the dehydration process occurred at a higher initial temperature than that determined by TGA analysis.

### 3.3. Water Absorption Capacity

WAC is an important feature of food ingredients and additives since it influences moisture content and quality attributes of the products [51]. It is especially valid for meat and milk processing where proteins and fibers are the major components used to achieve desired water holding capacity [52–54]. The production of flour-based food is another industrial sector with high demand for fiber concentrates [55]. They are often added to whole grain bread, noodles, and biscuits to achieve desired organoleptic properties and health benefits. Currently, there is a wide pool of plant fiber concentrates prepared from various agri-food waste or by-products [56–58]. Regardless, any of them, including DSM, is a valuable step toward the efficient use of natural resources and a decrease in food-processing waste. Fiber concentrates, prepared from different plant sources, are distinguished by their mode of preparation and chemical composition which impacts their functional properties and direct comparison may not be accurate. The DSM was obtained after several steps with specific conditions (Figure 1), but a relative comparison of this product to similar ones reported in literature could facilitate any potential application, optimization, or modification of the material.

**Figure 4:**
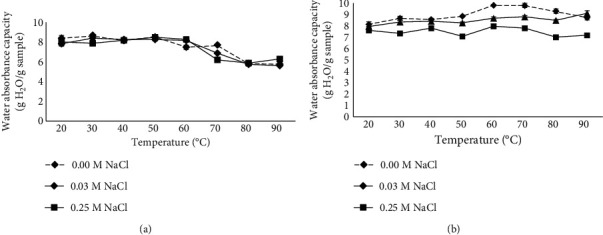
Influence of temperature and NaCl concentrations on water absorption capacity of deproteinized ethanol-treated sunflower meal at pH 6 (a) and pH 7 (b).

DSM exhibited high WAC which was dependent on both pH and NaCl concentration (Table 5). At the low acidic pH range (3 and 4), the WAC of the DSM was the lowest, while at pH 6 and 7, the WAC reached the highest values for all NaCl concentrations studied. This was the reason to further evaluate the influence of temperature on the WAC of the sample at these specific pH values (Figure 4).

Except for pH 5, the WAC of the DSM without the addition of NaCl was either higher or the same as the WAC of the DSM with NaCl supplementation, 0.03 M or 0.25 M. Compared to deproteinized rapeseed meal, evaluated by Georgiev et al. [13], the WAC of the DSM was approximately 1.5 fold higher under all studied conditions. This might be due to differences in the biochemical composition of the two samples and/or specific interactions and synergisms of components of the product. The DSM contained higher protein but lower dietary fiber amounts than the deproteinized rapeseed meal. By evaluating the WAC of various proteins and polysaccharides, used as additives in the meat industry, Köhn et al. [51] established that the average WAC of the proteins in NaCl concentrations up to 6% was higher than the ones of the polysaccharides. The result was though opposite for the experiments performed with 8% NaCl. Compared to sunflower oilcake, studied by Petraru et al. [35], the WAC of the DSM was approximately 3 fold higher. This might be due to the high amount of oil contained in the cakes. While the oil content in the DSM was estimated at 2.38% (Table 1), the studied oilcake contained 14-15% oil. The hydrophobic nature of vegetable oil does not favor interactions with water molecules and may adversely affect the WAC of materials. Under all studied conditions, the WAC of the DSM was lower than all types of papaya dietary fiber concentrates [47] but higher than that determined for wheat bran fiber (2.9-5.8 mL/g) by Jacobs et al. [59]. It was also higher than the water retention capacity of the fiber concentrates from skins and stems but similar to lees which are by-products of the wine-making industry [60].

Temperature increases influenced the WAC of DSM differently depending on pH (Figure 4). At pH 6 (Figure 4(a)), enhancement of temperature above 60°C decreased the WAC of the DSM with and without NaCl supplementation. At pH 7, the elevation of temperature increased the WAC from 8.13 g H_2_O/g sample at 20°C to 9.80 g H_2_O/g sample at 60°C (Figure 4(b)). Further increase in the temperature diminished the WAC of the DSM. The increase of temperature slightly increased the WAC of the DSM containing 0.03 M NaCl, which, however, remained lower than that of the DSM without NaCl addition. The higher level of NaCl (0.25 M) did not augment the WAC of the DSM regardless of temperature increase. Meaning, the mixed composition of the DSM and the observed results are difficult to explain. They might be due to the type and structure of ionized groups at different pH values and their interactions with NaCl ions from one side and water molecules from the other. The results imply a potential application of the fiber concentrate as a valuable additive in the food industry. Modulation of the WAC by pH and NaCl concentrations is another benefit that allows the achievement of a product with desired characteristics. The versatile results obtained with the two boundary concentrations of NaCl, 0.03 M and 0.25 M, suggest a need for further elucidation of this factor by exploring the effect of intermediate concentrations.

## 4. Conclusions

DSM was characterized as a valuable ingredient/additive for the food industry. It contained a relatively high amount of proteins and dietary fibers, which along with the remaining compounds defined it as a fiber concentrate. The DSM exhibited high WAC which was the most pronounced at pH 7. At this specific pH, the increase of temperature up to 60°C slightly enhanced the WAC of the DSM. The results demonstrated that material, generally discarded as waste after protein isolation for sunflower meal, could turn into a product with useful functional characteristics. Being a by-product by itself, the upcycling of the sunflower meal to multiple useful products (protein isolate and fiber concentrate) leads to better utilization of the plant material and natural resources as a whole.

## Figures and Tables

**Table 1:** Biochemical composition of deproteinized ethanol-treated sunflower meal.

Component	Content^∗^ [%]
Protein	19.88 ± 0.44
Ash	11.73 ± 0.62
Total lipids	2.38 ± 0.36
Total dietary fibers (i) Insoluble (ii) Soluble	61.06 ± 0.61 53.09 ± 0.48 4.59 ± 0.10
Total phenols	0.04 ± 0.00

^∗^Contents are calculated on a dry matter basis, 94.44 ± 0.14%.

**Table 2:** Fractional profile of alcohol-insoluble deproteinized ethanol-treated sunflower meal.

Component	Content [g/100 g]
Total (noncellulosic) carbohydrates	14.86 ± 0.16
Uronic acids	2.28 ± 0.10
Cellulose	30.87 ± 0.22
Lignin	21.79 ± 0.18

**Table 3:** Monosaccharide composition of deproteinized ethanol-treated sunflower meal.

Sample	Content (*μ*g/mg)								
	GlcA	GalA	Glc	Gal	Rha	Ara	Fuc	Xyl	Man
	1.83 ± 0.11	17.57 ± 2.87	180.49 ± 2.69	35.82 ± 1.12	2.28 ± 0.54	21.47 ± 1.47	6.57 ± 0.41	98.14 ± 0.15	29.61 ± 1.36

GlcA, D-Glucuronic acid; GalA, D-Galacturonic acid; Glc, D-glucose; Gal, D-galactose; Rha, D-rhamnose; Ara, D-arabinose; Fuc, D-fucose; Xyl, D-xylose; Man, D-mannose.

**Table 4:** Selected microelements and heavy metal contents of deproteinized ethanol-treated sunflower meal.

Component	Content [mg/kg]
Copper (Cu)	31.87
Iron (Fe)	133.29
Manganese (Mn)	31.87
Selenium (Se)	0.03
Zinc (Zn)	201.56

Lead (Pb)	<0.10
Cadmium (Cd)	<0.01

**Table 5:** Water absorption capacity of deproteinized ethanol-treated sunflower meal at different pH and NaCl concentrations.

pH	Water absorption capacity [g H_2_O/g sample]		
	0.00 M NaCl	0.03 M NaCl	0.25 M NaCl
3	6.05 ± 0.44^*b*,*A*^	6.30 ± 0.30^c,A^	5.48 ± 0.18^*c*,*A*^
4	6.98 ± 0.38^*b*,*A*^	7.55 ± 0.23^a,A^	5.95 ± 0.38^*c*,*B*^
5	7.24 ± 0.38^*b*,*B*^	7.86 ± 0.02^a,A^	7.52 ± 0.17^*a*,*B*^
6	8.39 ± 0.29^*a*,*A*^	7.84 ± 0.21^a,A^	8.02 ± 0.42^a,A^
7	8.13 ± 0.22^*a*,*A*^	7.93 ± 0.01^a,A^	7.59 ± 0.15^*a*,*AB*^
8	8.30 ± 0.15^*a*,*A*^	7.94 ± 0.26^a,A^	7.13 ± 0.11^*b*,*B*^
9	8.36 ± 0.38^*a*,*A*^	7.26 ± 0.24^b,B^	7.02 ± 0.15^*b*,*B*^
10	8.35 ± 0.18^*a*,*A*^	7.14 ± 0.13^*b*,*B*^	7.05 ± 0.07^*b*,*B*^

^a-c^ means in a column with different lowercase superscripts differ significantly (*p* < 0.05). ^A-B^ means in a row with different uppercase superscripts differ significantly (*p* < 0.05).

## Data Availability

All data are included in the manuscript.
